# Validation of the KIDSCREEN-27 Health-Related Quality of Life Questionnaire in a Sample of Mexican Adolescents

**DOI:** 10.3390/bs16050663

**Published:** 2026-04-28

**Authors:** Adalberto Muñoz-Márquez, Rodrigo Vargas-Salomón, Luis Manuel Blanco-Donoso, Rosa Martha Meda-Lara, Pedro Juárez-Rodríguez

**Affiliations:** 1 Centro de Evaluación e Investigación Psicológica, Departamento de Psicología Básica, Centro Universitario de Ciencias de la Salud, Universidad de Guadalajara, Guadalajara 44340, Mexico; adalberto.munoz6865@alumnos.udg.mx (A.M.-M.); rosa.meda@academicos.udg.mx (R.M.M.-L.); 2 Doctorado en Psicología de la Salud, Departamento de Psicología Básica, Centro Universitario de Ciencias de la Salud, Universidad de Guadalajara, Guadalajara 44340, Mexico; 3 Departamento de Derecho Privado, Centro Universitario de Ciencias Sociales y Humanidades, Universidad de Guadalajara, Guadalajara 45132, Mexico; rodrigo.vargas0848@academicos.udg.mx; 4 Departamento de Psicología Biológica y de la Salud, Facultad de Psicología, Universidad Autónoma de Madrid, 28049 Madrid, Spain; luismanuel.blanco@uam.es

**Keywords:** KIDSCREEN, quality of life, adolescents, psychometric properties, validation

## Abstract

**Background**: Health-related quality of life (HRQoL) in adolescents reflects their perception of physical, psychological, and social well-being within a specific cultural context, considering developmental stage and individual differences. The KIDSCREEN-27 is a self-report instrument designed to assess HRQoL in children and adolescents, with demonstrated validity and reliability in international samples. **Objective**: To examine the psychometric properties (i.e., reliability, construct validity, convergent and discriminant validity, and measurement invariance) of the KIDSCREEN-27 questionnaire in a sample of Mexican adolescents. **Method**: A cross-sectional study was conducted with a sample of 1124 Mexican adolescents aged 10–17 years (M = 13.37, SD = 1.08; 53.5% female; 83.6% secondary education) obtained through non-probabilistic convenience sampling. Reliability (Cronbach’s α, McDonald’s ω), structural validity through exploratory (AFE) and confirmatory factor analyses (CFA), measurement invariance by gender, and convergent and discriminant validity via correlations with self-esteem, well-being, stress, and anxiety–depressive symptoms were evaluated. **Results**: Analyses showed strong internal consistency (α = 0.912, ω = 0.914). EFA supported a five-dimensional structure. CFA showed an optimal fit after including specific covariances (*χ*^2^/*df* = 3.62, RMSEA = 0.048, CFI = 0.929, TLI = 0.919, SRMR = 0.043). Metric and scalar gender invariance were supported. Positive correlations emerged with well-being (*r* = 0.76, *p* < 0.01), self-esteem (*r* = 0.64, *p* < 0.01), and satisfaction with life (*r* = 0.52, *p* < 0.01), and negative correlations with stress (*r* = −0.61, *p* < 0.01), academic stress (*r* = −0.32, *p* < 0.01) and anxiety–depressive symptomatology (*r* = −0.53, *p* < 0.01), providing evidence of convergent and discriminant validity. **Conclusions**: The KIDSCREEN-27 demonstrated adequate psychometric properties, supporting its use among Mexican adolescents, enabling the identification of well-being needs, monitoring of interventions, informed decision-making in health and educational practice and supporting cross-cultural comparisons of adolescent well-being.

## 1. Introduction

According to the World Health Organization (WHO, 1948), health is defined as a state of complete physical, mental, and social well-being, and not merely the absence of disease. This multidimensional conceptualization lays the epistemological foundation for the contemporary understanding of quality of life (QoL), which is grounded in the subjective perception of well-being across these same domains. Accordingly, this perception encompasses physical, social, mental, and emotional well-being, along with the capacity to engage in meaningful daily activities. When explicitly considered in relation to health status, this construct is operationalized as Health-Related Quality of Life (HRQoL) (The WHOQOL Group, 1998).

Attempts to encompass the complexity of quality of life have generated ambiguities in its definition and measurement, given the differences in conceptual approaches and assessment criteria employed (Haraldstad et al., 2019; Karimi & Brazier, 2016; Moons et al., 2006; Prutkin & Feinstein, 2002). To conceptually delimit HRQoL, we chose the definition of quality of life proposed by the WHO as a starting point; namely, “an individual’s perception of their position in life in the context of the culture and value systems in which they live and in relation to their goals, expectations, standards, and concerns” (The WHOQOL Group, 1993). This notion is particularly relevant during adolescence, a transitional stage of the life cycle characterized by an intensified search for identity and the emotional changes inherent to this developmental period (Figueroa et al., 2018), in which individuals are particularly sensitive to the influence of society and their environment (Moore Heslin & McNulty, 2023).

Building upon this conceptual foundation, HRQoL is conceived as a multidimensional construct that integrates objective and subjective factors linked to well-being and functionality. In line with this approach, several studies have identified dimensions consistent with this conception, such as psychological well-being (Castañeda Gallego et al., 2017), economic stability and material resources in the family environment (Park & Look, 2018), interpersonal relationships and social support (Lezhnieva et al., 2018; Stevens et al., 2018), autonomy and independent functioning (da Silva & Cavalcante, 2019; Vincent-Onabajo & Shaphant, 2019), and occupational and academic performance (Brekke et al., 2019). Family support has also been identified as a fundamental component of perceived HRQoL during adolescence (Roberts & Ishler, 2018).

The WHO defines adolescence as the period of growth and development that occurs after childhood and before adulthood, between the ages of 10 and 19. This stage is one of the most important transitions in human life, given the rapid pace of growth and change. In particular, puberty marks the beginning of adolescence, while other biological processes condition the passage through this crucial stage. Although its biological determinants are universal, the duration and characteristics of adolescence vary depending on the cultural and socioeconomic context (PAHO, 2017).

Measuring HRQoL in children and adolescents is complex due to the natural process of development and progress through the different stages of physical and intellectual maturation. For this reason, perceptions of satisfaction with health and well-being may vary (Rajmil & Herdman, 2019). To perform an accurate assessment in children and adolescents, specific elements must be considered, such as developmental stage and the school and family context, as well as whether the instrument is generic or specific to a particular condition, the age group it is aimed at, whether it is self-reported or proxy-reported, and its psychometric properties (Barnes & Jenney, 2002).

Important and diverse domains have been proposed that should be included in instruments for measuring QoL regardless of cultural context, such as psychological health, physical health, level of independence, social relationships with parents and peers, environment, and personal/spiritual beliefs (Jirojanakul & Skevington, 2000). In this broad context, the Kidscreen questionnaire emerged as an instrument of the Kidscreen Project promoted by the European Union (Ravens-Sieberer et al., 2001), whose main objective was to produce a reliable self-report measure of HRQoL for healthy or ill children and adolescents with an emphasis on cultural issues.

The 52-item version KIDSCREEN-52 has demonstrated methodological and conceptual strength, covering 10 dimensions of the HRQoL construct (physical well-being, psychological well-being, mood, self-perception, autonomy, relationship with parents and family life, economic resources, social and peer support, school environment, social acceptance) (Ravens-Sieberer et al., 2008). Adaptation studies of this version of the instrument have consistently shown good internal consistency, with Cronbach’s alpha values ranging from 0.77 to 0.89, as well as good temporal stability, making it a reliable tool for both epidemiological research and longitudinal studies (Haraldstad et al., 2011; Berra et al., 2013; Hidalgo Rasmussen et al., 2014; Galán Cuevas & Díaz García, 2021). Nevertheless, according to some authors, its main weakness is its length, which can place a heavy burden on children and adolescents, limiting its practicality in routine clinical settings or in populations with fatigue or attention difficulties (Ravens-Sieberer et al., 2007; Robitail et al., 2007).

The 27-item version, KIDSCREEN-27, emerges as a balanced alternative, maintaining psychometric robustness and focus on adolescents, but with a reduced length of five dimensions (physical well-being, psychological well-being, autonomy and relationship with parents, social and peer support, school environment) and strategic optimization of the same theoretical construct (Molina et al., 2014; Vélez et al., 2016). Its superior usefulness in applied contexts lies in the fact that it maintains the cross-cultural validity and internal consistency of the essential dimensions of adolescence (physical, psychological, social, and school). It reduces cognitive and time burdens, preventing individual fatigue and making the KIDSCREEN-27 a brief and useful tool, maximizing data quality by minimizing the risk of random responses or questionnaire abandonment (The KIDSCREEN Group Europe, 2006).

In Argentina, Brazil, and Chile, validations of the KIDSCREEN-27 have been carried out with psychometric properties similar to those of the European version. Urzúa et al. (2009), in their validation in Chilean adolescents, reported an internal consistency greater than 0.70 for all dimensions and confirmed the five-factor structure through confirmatory factor analysis (CFA). Similarly, the Colombian version demonstrated adequate psychometric properties in children and adolescents in Medellín, with internal consistency ranging from 0.71 to 0.82 across its five dimensions (Quintero et al., 2011).

Molina et al. (2014), in their validation study of the KIDSCREEN-27 in Chilean adolescents, reported an internal consistency of 0.89 for the entire instrument and confirmed the five-dimension structure through CFA. On the other hand, the Brazilian version demonstrated adequate psychometric properties in adolescents, with reproducibility, internal consistency, and construct validity values that confirm its usefulness in that population (da Silveira et al., 2021).

In Mexico, efforts to adapt the KIDSCREEN have focused on its 52-item version, yielding satisfactory psychometric results and supporting the use of this scale in the Mexican population (Galán Cuevas & Díaz García, 2021; Hidalgo Rasmussen et al., 2014); however, the KIDSCREEN-27 has not previously been validated in the Mexican context. This gap is significant, as the absence of a validated brief instrument with strong methodological and conceptual foundations limits the feasibility of assessing health-related quality of life (HRQoL) in Mexican adolescents within clinical, educational, and community settings. Longer instruments, while robust, may pose challenges in terms of administration time, respondent burden, and applicability in large-scale screening or routine evaluations. The availability of a validated shorter version, such as the KIDSCREEN-27, would enable more efficient measurement without compromising methodological quality, facilitating its integration into contexts where brevity and practical utility are essential.

### Aims

In light of the above, the primary objective of this study was to evaluate the psychometric properties (i.e., reliability, construct validity, convergent and discriminant validity, and measurement invariance) of the KIDSCREEN-27 for assessing health-related quality of life (HRQoL) among Mexican adolescents. To achieve this aim, the following specific objectives were established:

- To verify the KIDSCREEN-27 factorial structure through confirmatory factor analysis.
- To assess the measurement invariance of the KIDSCREEN-27 across sex.
- To estimate the reliability of the KIDSCREEN-27 using Cronbach’s alpha and McDonald’s omega coefficients.
- To examine the convergent validity of the KIDSCREEN-27 through a correlation analysis between HRQoL and satisfaction with life, self-esteem, and well-being.
- To evaluate discriminant validity by analyzing the correlations between the KIDSCREEN-27 and stress, academic stress, and symptoms of depression and anxiety.

## 2. Materials and Methods

### 2.1. Study Design and Procedures

We conducted a cross-sectional validation study involving relatively healthy Mexican adolescents. The sample was obtained through non-probabilistic convenience sampling in six public institutions of basic education (secondary school) and upper secondary education (high school) within the metropolitan area of the state of Jalisco, Mexico. Data were collected between September and November 2024. The survey was administered in person during class time using paper and pencil, as well as virtually using mobile devices with the assistance of a trained administrator, a member of the research team. Both the pencil-and-paper and mobile administrations were conducted in a guided manner, using identical instructions and similar time frames, thereby minimizing bias. The decision to employ a dual mode of administration was driven by practical considerations of accessibility and the need to reach adolescents in vulnerable situations who lack access to mobile devices. The participating institutions facilitated the recruitment process by extending invitations to parents. During an informational meeting conducted by members of the research team, informed consent forms were distributed to parents along with assent forms for minors. These documents were taken home to allow families to consider their voluntary participation in the study, after which each classroom teacher collected the signed informed consent forms from parents and the assent forms from minors prior to participation. All parents and participants received information about the study from the research team. In total, 1263 surveys were administered. Adolescents with special educational needs were excluded from the study to ensure response comparability and to mitigate potential difficulties in comprehending the questionnaire (n = 5). Additionally, incomplete questionnaires and those containing response errors were removed (n = 134).

### 2.2. Instruments

Ad hoc questionnaire on basic sociodemographic data, including age and gender.

27-item KIDSCREEN questionnaire (The KIDSCREEN Group Europe, 2006): This is the shortest version of the KIDSCREEN questionnaires in Spanish. It consists of 27 items distributed across five dimensions: Physical well-being (e.g., Have you felt well and fit?); Psychological well-being (e.g., Have you enjoyed life? Autonomy and parents (e.g., Have you had enough time for yourself?); Social and peer support (e.g., Have you spent time with your friends?); and School environment (e.g., Have you felt happy at school?). It uses a 5-point Likert-type response scale ranging from 1 = never to 5 = always. All items reference a recall period of the previous week. Raw scores are obtained by summing the item responses within each dimension. Items formulated with negative wording (items 9, 10, and 11) are reverse-coded to ensure consistency with the direction of positively formulated items. Dimension scores are subsequently transformed to a standardized T-score metric (range 0–100), with higher scores indicating better health-related quality of life (da Silveira et al., 2021; The KIDSCREEN Group Europe, 2006). Adaptations in Latin America have demonstrated adequate internal consistency, with a Cronbach’s alpha of 0.89 (Molina et al., 2014). In the present study, Cronbach’s alpha coefficient was α = 0.91.

Rosenberg Self-Esteem Scale (RSES) (Rosenberg, 1965): This instrument is a widely utilised and validated 10-item questionnaire designed to assess self-esteem by capturing both positive and negative self-perceptions (e.g., “In general, I am satisfied with myself”). Responses are recorded on a 4-point Likert scale ranging from 1 (strongly agree) to 4 (strongly disagree), with several items reverse-scored. Higher total scores reflect greater levels of self-esteem. The Mexican adaptation of the RSES has shown acceptable internal consistency, with a Cronbach’s alpha of 0.79 (Jurado et al., 2015). In the current study, Cronbach’s alpha coefficient was 0.77.

Satisfaction with Life Scale (SWLS) (Diener et al., 1985): The Spanish adaptation (Atienza et al., 2000), consisting of five items, was used in this study with minor modifications to improve clarity in the Mexican context. For example, the first item, originally expressed as “In most respects, my life is close to my ideal,” was adapted to “My life, in almost all respects, meets my aspirations.” The other items on the SWLS were as follows: “My living conditions are excellent,” “I am satisfied with my life,” “So far in my life, I have achieved things that were important to me,” and “If I were born again, I would change many things in my life” (reverse item). Responses were obtained using a 7-point Likert scale, with options ranging from 1 (strongly disagree) to 7 (strongly agree). Higher scores reflected greater levels of life satisfaction. Prior research in Spanish populations has reported Cronbach’s alpha coefficients ranging from 0.79 to 0.89 (Amezquita et al., 2008; Atienza et al., 2000). In the current study, Cronbach’s alpha coefficient was 0.82.

WHO Well-Being Index (WHO-5) (WHO, 1998): This is a brief, generic questionnaire designed to assess subjective psychological well-being. It uses a 6-point Likert scale, based on frequency over the last 14 days (0 = Never, 1 = Sometimes, 2 = Less than half the time, 3 = More than half the time, 4 = Most of the time, and 5 = All the time). A higher total score indicates greater well-being (e.g., I have felt cheerful and in good spirits). It has demonstrated internal consistency in a Mexican student population, with Cronbach’s alpha and McDonald’s omega values of 0.90 (Macias Becerril et al., 2025). In the present study, Cronbach’s alpha coefficient was 0.87.

Perceived Stress Scale (PSS-10) (Cohen et al., 1983): It consists of 10 items and focuses on the perception of stress in the last month, using a 5-point response scale (0 = “never,” 1 = “almost never,” 2 = “sometimes,” 3 = “quite often,” 4 = “very often”). PSS-10 scores were obtained by adding the positive items and reversing the scores of the items designed to assess the absence of stress (e.g., Have you felt that you are able to control the important things in your life?), with higher scores indicating higher levels of perceived stress (Cohen et al., 1997). Its use in Mexican samples reports a Cronbach’s alpha coefficient of 0.83 (Flores-Torres et al., 2021). In the present study, Cronbach’s alpha coefficient was 0.72.

Academic Stressors Subscale (Barraza, 2007a): The Academic Stressors Subscale of the SISCO Inventory (Symptoms, Stressors, and Coping Strategies), developed to assess academic stress in the student population. It includes eight Likert-type items with five categorical values (1 = never, 2 = rarely, 3 = sometimes, 4 = almost always, and 5 = always) that allow for identifying the frequency with which environmental demands are assessed as stressors (e.g., competition with classmates). It is part of a broader inventory and has shown a reliability of 0.90 in previous studies (Barraza, 2007b). In the present study, Cronbach’s alpha coefficient was 0.78.

Patient Health Questionnaire (PHQ-4) (Kroenke et al., 2009): This brief and widely used self-report measure is designed to screen for core symptoms of both depression and anxiety. It consists of four items that capture essential aspects of these conditions. Responses are provided on a 4-point Likert scale, indicating the frequency of symptoms experienced over the previous two weeks. In the Mexican population, the instrument has demonstrated adequate internal consistency for both the anxiety subscale (ω = 0.78) and the depression subscale (ω = 0.82) (Avalos-Salcedo et al., 2025). In the current study, Cronbach’s alpha coefficient was 0.77.

### 2.3. Statistical Analysis

The data were analyzed as categorical variables using frequency distributions. The normality of the variables was assessed using the Kolmogorov–Smirnov test, and those that did not meet this assumption were subjected to analysis using nonparametric tests. Normality was also assessed by reviewing the values of asymmetry and kurtosis. In social research, distributions with values between −2 and +2 are considered approximately normal and suitable for statistical analyses such as regression or factor analysis (Byrne, 2016; Hair et al., 2019; Kline, 2023). Internal consistency was determined using item–item and item–total correlations as well as Cronbach’s alpha and McDonald’s omega coefficients (Green & Yang, 2009; McDonald, 1999). The omega coefficient was chosen to estimate the reliability of multidimensional instruments with items of heterogeneous psychometric characteristics (Hayes & Coutts, 2020). Evidence of construct validity was obtained by evaluating the Kaiser–Meyer–Olkin (KMO) measure of sample adequacy and using Bartlett’s sphericity test to analyze correlations appropriate for factor analysis, following the criteria of Hair et al. (2019). The stability of the factorial structure was verified with exploratory factor analysis (EFA), considering possible changes in response patterns due to contextual, sociocultural, and population characteristics. The extraction method employed was maximum likelihood and direct oblimin oblique rotation. CFA was performed, and the goodness of fit of the model was examined using the Root Mean Square Error of Approximation (RMSEA) (Hu & Bentler, 1999; West et al., 2012), the Comparative Fit Index (CFI), the Normed Fit Index (NFI), the Tucker–Lewis Index (TLI), and the Residual-Based Absolute Fit Index (SRMR) (Brown, 2015; Kline, 2023). It was assumed that the relationship between the five dimensions was correlated. The overall fit of the model was assessed using the chi-square test (*χ*^2^) and normed chi-square (*χ*^2^/*df*). Conventional criteria were used to evaluate the fit: SRMR < 0.08, RMSEA < 0.06, and CFI/TLI > 0.90 (Hu & Bentler, 1999). To obtain evidence of the convergent and discriminant validity of the KIDSCREEN-27, correlation analyses were performed for the KIDSCREEN-27 with measures of subjective well-being, life satisfaction, and self-esteem (convergent validity), all directly related to quality of life (Mokkink et al., 2018), as well as measures of anxiety, depression, stress, and academic stress (discriminant validity), given that these constructs are theoretically opposed to quality of life. To assess the measurement invariance of the KIDSCREEN-27, an invariance analysis by gender was performed. This procedure was carried out using a hierarchical sequence of multigroup confirmatory factor analyses (MG-CFAs), in which three levels of standard invariance were progressively examined and compared: configural, metric, and scalar. The data obtained were analyzed using IBM SPSS Statistics v.31 software (IBM Corp, Armonk, NY, USA), and the CFA models were estimated using IBM AMOS v.31 (IBM Corp, Armonk, NY, USA) to compare the power and fit of the models. Values of α < 0.05 were considered statistically significant.

### 2.4. Ethical Considerations

The research project was evaluated and approved by the Ethics and Research Committee of the University Center for Health Science of the Universidad de Guadalajara (Mexico), with folio number CI-06524. The study was conducted according to the guidelines of the Declaration of Helsinki. All participants included in the study voluntarily provided their informed consent after reading the purposes of the study. Data are stored in a locked and password-protected computer under the principal investigator’s safekeeping to maintain confidentiality.

## 3. Results

### 3.1. Characterization of the Adolescent Sample

A total of 1124 adolescents from Mexico participated in this study. The proportion of male and female participants was similar (46.5% and 53.5%, respectively), and their ages ranged from 11 to 17 years (M = 13.37, SD = 1.08), with the highest concentration between 13 and 14 years (59.9%). Most participants were enrolled in preparatory high school (83.6%) (see Table 1).

### 3.2. Data Distribution and Internal Consistency

The distribution of raw data at the item level showed values of skewness and kurtosis within ±2.0, indicating normality (see Table 2).

The distribution of data for each of the dimensions showed values of skewness and kurtosis ranging from −0.265 to −0.944 and −0.386 to 0.778, respectively, within ±2.0, indicating normality. In accordance with the distribution of data, parametric tests and factor analyses were performed.

### 3.3. Exploratory Factor Analysis (EFA)

Although the KIDSCREEN-27 has solid evidence of structural validity in European, Eastern, and American samples (Li et al., 2024; Ardelean et al., 2022; da Silveira et al., 2021; Urzúa et al., 2009; Quintero et al., 2011), prior to CFA, we aimed to confirm the latent structure of five dimensions. The extraction method employed was maximum likelihood and direct oblimin oblique rotation. The following conventional criteria were used for evaluation: KMO ≥ 0.80 and Bartlett’s test of sphericity of *p* < 0.05 (Hair et al., 2019). The suitability of the data for factor analysis was confirmed by an excellent KMO measure (0.92) and a significant Bartlett’s sphericity test (*χ*^2^(351) = 11,628.86, *p* < 0.01). The CFA extracted five factors that explain 55.13% of the variance before rotation and 46.01% of the total variance. The variance explained by each factor after rotation ranged from 3.23% to 21.16%, redistributing the variance among the five factors.

### 3.4. Confirmatory Factor Analysis

The theoretical structure of the five dimensions of the KIDSCREEN-27 was evaluated using CFA with all the data. Conventional criteria were used to evaluate the fit: SRMR < 0.08, RMSEA < 0.06, and CFI/TLI > 0.90 (Hu & Bentler, 1999). The initial model did not achieve an optimal fit according to conventional criteria. Although the SRMR (0.058) indicated an acceptable fit, the RMSEA (0.070) showed a moderate approximation error, and the incremental indices CFI (0.847) and TLI (0.829), together with a high normalized chi-square (6.53), evidenced a significant mismatch between the proposed structure and the empirical data. Based on these results, we examined the modification indices (MIs), given that items 9, 10, and 11 of dimension two assess similar aspects of unpleasant feelings and emotions, and items 18 and 19 of dimension three share a focus on economic autonomy. Covariances were established between their error terms to capture this source of common variation not explained by the latent factor. These covariances were incorporated to generate a new model.

**Figure 1:**
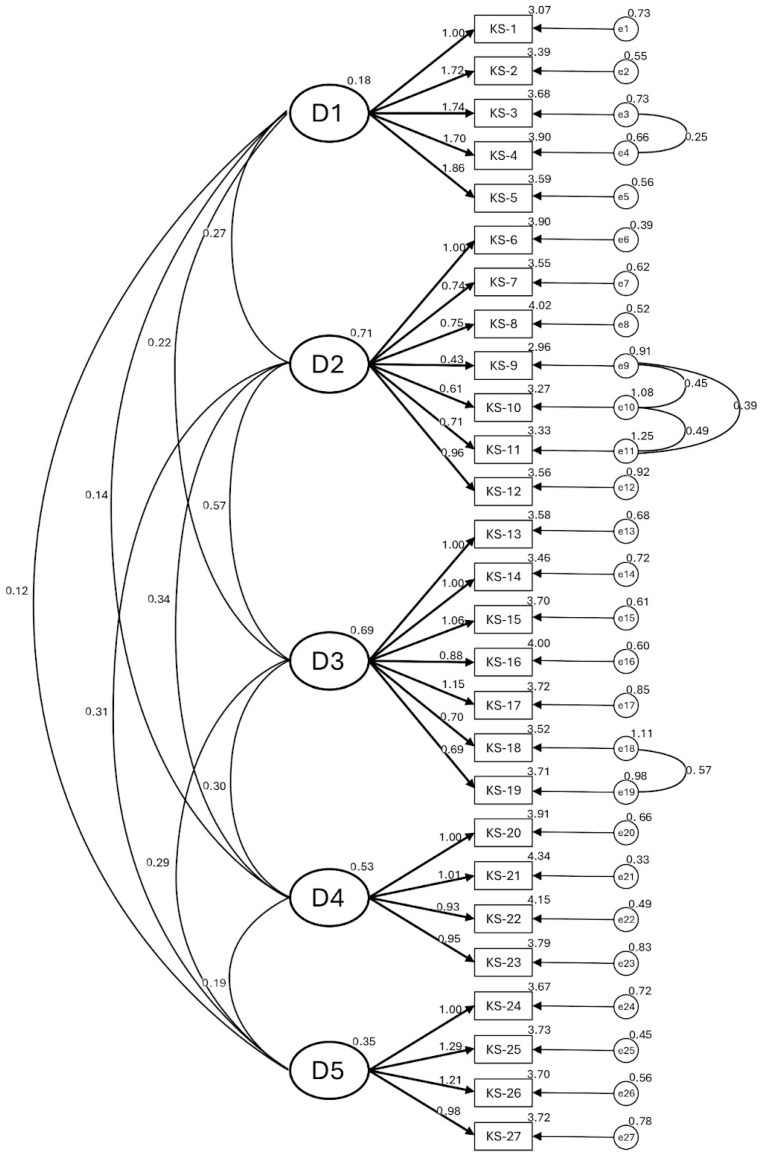
Five-factor structure model of the Mexican version of the KIDSCREEN-27. D1: Physical Well-Being; D2: Psychological Well-Being; D3: Autonomy and Parents; D4: Social and Peer Support; D5: School Environment.

After this analysis, the model was modeler-specified using CFA, revealing a progressive and substantial improvement in the fit indices. Starting from the initial model (M1) with inadequate fit, the sequential incorporation of covariances between item error terms (within-dimension, specifically between pairs 9 and 10, 10 and 11, 9 and 11, 18 and 19, and finally 3 and 4) resulted in incremental improvements in all indicators until the final model (M6) with optimal fit was achieved: *χ*^2^/*df* = 3.62, RMSEA = 0.048, CFI = 0.929, TLI = 0.919, and SRMR = 0.043. All these values meet the criteria for acceptable fit (*χ*^2^/*df* < 5; RMSEA < 0.06; CFI/TLI > 0.90; SRMR < 0.08), strongly supporting the factor structure of the KIDSCREEN-27 after introducing the specified modifications (see Table 3). The CFA results corroborate the factorial structure of the KIDSCREEN-27 among Mexican adolescents (see Figure 1).

Conventional criteria were used to interpret the coefficients of internal consistency: α > 0.70 are considered acceptable, >80, very good, >0.90 excellent (Streiner, 2003); ω > 0.70 are considered acceptable, >80, very good, >0.90 excellent (McDonald, 1999). The internal consistency of the KIDSCREEN-27 dimensions showed adequate indices. Cronbach’s alpha coefficients for the dimensions ranged from α = 0.727 to 0.845, with an overall α = 0.912 indicating adequate homogeneity among the items. Likewise, McDonald’s omega coefficients for the dimensions ranged from ω = 0.735 to 0.845, with an overall ω = 0.914, providing evidence of the model’s reliability when considering the actual factor loadings of the items (see Table 4). These results highlight the adequate internal consistency of the instrument in the sample of Mexican adolescents.

### 3.5. Convergent Validity

To assess convergent validity, correlations between KIDSCREEN-27 scores and theoretically related construct measures were analyzed. Analyses using Pearson’s correlation coefficient revealed positive, statistically significant, and moderate-to-strong correlations between the total HRQoL score and measures of subjective well-being (*r* = 0.76, *p* < 0.01), satisfaction with life (*r* = 0.52, *p* < 0.01), and self-esteem (*r* = 0.64, *p* < 0.01) (see Table 5). This pattern was consistently replicated across the five dimensions of the instrument, with the psychological well-being dimension showing the strongest associations. These results support the convergent validity of the KIDSCREEN-27 in relation to other similar theoretical constructs among Mexican adolescents.

### 3.6. Discriminant Validity

To examine discriminant validity, correlations between the KIDSCREEN-27 and measures of theoretically opposite constructs were analyzed. Analyses using Pearson’s correlation coefficient revealed statistically significant inverse correlations of moderate-to-strong magnitude between the total HRQoL score and measures of perceived stress (*r* = −0.61, *p* < 0.01), anxious and depressive symptomatology (*r* = −0.53, *p* < 0.01), and academic stress (*r* = −0.32, *p* < 0.01) (see Table 6). This pattern of inverse correlations was consistent, supporting the instrument’s ability to discriminate between theoretically opposed constructs. These results support the discriminant validity of the KIDSCREEN-27 in relation to theoretical constructs opposed to HRQoL, such as psychological distress (stress, anxiety symptoms, and depressive symptoms), among Mexican adolescents.

### 3.7. Multigroup Confirmatory Factor Analysis

Factor invariance across groups according to sex was evaluated in the five-dimensional structure of the KIDSCREEN-27. A hierarchical sequence of four models of configuration, metric, scalar, and strict measurement invariance was applied (Byrne, 2016). Each subsequent model was tested only if an adequate fit was achieved in the previous one. All models showed an excellent fit (RMSEA = 0.040, CFI > 0.890) (see Table 7). Following the criterion of ΔCFI < 0.01, metric (ΔCFI = 0.002) and scalar (ΔCFI = 0.001) invariance were accepted. Based on the overall data from the three criteria, the KIDSCREEN-27 shows invariance across sex.

## 4. Discussion

The objective of this study was to analyze the psychometric properties of the KIDSCREEN-27 and validate its use in a sample of Mexican adolescents, addressing the need for brief, reliable, and culturally relevant instruments for assessing HRQoL at this stage of development. The results obtained provide solid evidence that the Mexican version of the KIDSCREEN-27 is a valid and reliable instrument for this purpose, contributing significantly to the field of HRQoL assessment in the adolescent population in Mexico. The main contribution of this study lies in providing the first validation of this instrument in Mexican adolescents, expanding the availability of psychometrically robust tools beyond its extensive 52-item version, which has previously been validated in Mexico [EE-T1] (Hidalgo Rasmussen et al., 2014). The availability of a short version is especially relevant in educational and community settings, where time constraints and assessment load make it necessary to use concise instruments without compromising psychometric quality (Brooks et al., 2003). In this sense, the KIDSCREEN-27 is positioned as an efficient alternative for assessing HRQoL in population studies, prevention programs, and intervention evaluations (Ravens-Sieberer et al., 2007).

Specifically, the results of the reliability analysis showed high levels of internal consistency across all dimensions of the instrument. These values are consistent with—and, in some cases, slightly higher than—those reported in the original European version and in previous international validations of the KIDSCREEN-27 conducted in countries such as Argentina, Brazil, and Chile, where total reliability coefficients typically range between 0.85 and 0.90. At the dimensional level, the coefficients obtained are also comparable to those reported in European and Latin American samples (generally above 0.70), further supporting the robustness of the instrument across cultural contexts. Moreover, the use of the omega coefficient, particularly recommended for multidimensional instruments, reinforces the strength of this evidence and supports the internal stability of the scores obtained in the Mexican sample (Hayes & Coutts, 2020). The slightly higher overall reliability observed in this study may be associated with the relative homogeneity of the sample in terms of age and educational context, as well as with the strong inter-item correlations identified in certain domains.

The structural validity of the KIDSCREEN-27 in Mexican adolescents was supported by both EFA and CFA. The EFA indicated the expected five-factor solution, consistent with the original European model and studies conducted in Chile and other contexts. The initial CFA results, however, showed an inadequate fit, contrasting with some European and Chilean studies reporting acceptable indices without modifications. After model respecification, including the introduction of correlated error terms—particularly between items 9 and 11 (negative affect) and 18 and 19 (autonomy/economic resources)—the final model achieved optimal fit, consistent with recommended thresholds and comparable to best-fitting models internationally. These results confirm the five-dimension structure—physical well-being, psychological well-being, autonomy and parents, social and peer support, and school environment—demonstrating adequate representation of the HRQoL construct. Overall, these findings further support the structural validity of the KIDSCREEN-27 in Mexican adolescents and reinforce its comparability with international studies (Ravens-Sieberer et al., 2007). The need for correlated errors and minor deviations from the original structure, also observed in other Latin American validations such as Colombia (Quintero et al., 2011), may reflect cultural differences in the interpretation of emotional distress and autonomy, as well as contextual factors like family interdependence and socioeconomic variability, which can increase conceptual overlap between items.

A particularly relevant finding is the demonstration of metric and scalar invariance by gender, indicating that the instrument measures HRQoL equally in men and women. This evidence indicates that the differences observed in the scores can be interpreted as real differences in HRQoL levels and not as artifacts derived from measurement biases, strengthening the use of the KIDSCREEN-27 in comparative studies and analyses of gender inequalities in adolescence (Hambleton et al., 2005).

On the other hand, the positive and significant correlations between the dimensions of the KIDSCREEN-27 and variables such as subjective well-being, self-esteem, and life satisfaction, as well as the negative correlations with stress and anxiety–depressive symptoms, provide consistent evidence of convergent and discriminant validity (Vázquez & Hervás, 2008). Thus, these results confirm the sensitivity of the instrument in capturing the theoretical construct of HRQoL and its relationship with key indicators of mental health and psychological well-being in adolescence, a stage characterized by profound biopsychosocial changes and high emotional vulnerability (Steinberg, 2014).

The psychological well-being dimension showed the strongest correlations with measures of subjective well-being and self-esteem, suggesting that, in this context, psychological well-being is a central component of adolescents’ overall perceived quality of life. The high covariance observed among items assessing negative emotions indicates that Mexican adolescents may experience and conceptualize negative emotional states as a relatively unified construct, potentially influenced by cultural norms regarding emotional expression. The autonomy and parents dimension also demonstrated high internal consistency and positive correlations with overall well-being. This finding reflects that perceived parental support and autonomy are key factors for adolescent well-being and that, in the Mexican context, family interdependence and socioeconomic factors may strengthen the relationship between these variables and perceived quality of life. The physical well-being, social and peer support, and school environment dimensions showed moderate correlations with overall well-being and related constructs, suggesting that, although relevant, their influence may be secondary compared to psychological well-being and family relationships in this specific sample. This pattern may reflect particular educational and social contexts, where academic performance and peer relationships are important but do not dominate adolescents’ perceptions of quality of life. From an applied perspective, these results indicate that the KIDSCREEN-27 can be a useful tool for the comprehensive assessment of quality of life among Mexican adolescents, not only for research purposes but also for educational and health intervention programs.

Identifying the dimensions with the greatest impact—psychological well-being and family-related autonomy—highlights the potential for interventions aimed at promoting mental health and strengthening parental support to significantly enhance overall perceived quality of life in adolescents. In fact, validation of the KIDSCREEN-27 in a Mexican adolescent population provides a brief, reliable, and valid tool for monitoring HRQoL, allowing schools, health professionals, and researchers to identify risk areas early and implement more targeted preventive or supportive interventions. Its use can also facilitate informed decision-making in educational, clinical, and public health settings, supporting the design and evaluation of programs and interventions that are tailored to the specific needs of adolescents and sensitive to the sociocultural context. Overall, these findings underscore the practical relevance of the instrument not only for assessment but also for guiding the development of evidence-based strategies to enhance adolescent well-being. In addition, the relevance of these findings should be interpreted in light of the inherent complexity of measuring HRQoL in children and adolescents. The perception of well-being and health is influenced by the level of cognitive development, the sociocultural context, and the life experiences typical of this stage, which is why it is essential to have instruments with solid psychometric properties, such as the instrument evaluated in this study (Ravens-Sieberer et al., 2007; Hambleton et al., 2004).

This study has some limitations that should be acknowledged. Its cross-sectional design prevents examination of the temporal stability of the KIDSCREEN-27 and limits the ability to draw conclusions about changes in health-related quality of life over time, highlighting the need for future research to explore test–retest reliability and sensitivity to change in longitudinal or intervention studies. The use of non-probabilistic convenience sampling and the inclusion of relatively healthy, school-going adolescents restrict the generalizability of the findings to other populations, such as clinical samples, adolescents outside the formal education system, or those in socially vulnerable situations. Additionally, the exclusive reliance on self-report measures may introduce biases related to participants’ subjective perceptions, including social desirability or differential interpretation of items. Comparisons with other HRQoL instruments were also not conducted, which would have provided further evidence of concurrent validity and strengthened the performance evaluation of the KIDSCREEN-27. Despite these limitations, this study has important strengths, including a large sample size, balanced participation of male and female adolescents, the inclusion of participants from different educational levels, and the implementation of a culturally sensitive validation, which ensures the relevance and appropriateness of the instrument within the Mexican context (Hambleton et al., 2005). Moreover, the methodological rigor applied in the psychometric analyses reinforces the credibility of the findings and supports the use of the KIDSCREEN-27 for both research and applied purposes, while providing a foundation for future studies to address the identified limitations.

## 5. Conclusions

This study provides the first validation of the KIDSCREEN-27 for assessing HRQoL in Mexican adolescents, demonstrating that the short version is a psychometrically robust, reliable, and culturally appropriate instrument. Beyond confirming its validity and reliability, this work highlights the practical utility of KIDSCREEN-27 for educational, community, and clinical contexts, enabling the identification of well-being needs, the monitoring of interventions, and informed decision-making in health and educational practice. This study contributes to the scientific literature by expanding the availability of brief, culturally sensitive tools for HRQoL assessment, addressing an important gap in Latin American research, and supporting cross-cultural comparisons of adolescent well-being. Notably, evidence of factor invariance by gender, strong internal consistency, and adequate convergent and discriminant validity underscores the instrument’s applicability for rigorous research and professional use. Future research should examine the temporal stability of and sensitivity to changes in the KIDSCREEN-27, test its performance in clinical and vulnerable populations, and evaluate its invariance across different cultural or sociodemographic groups. Comparative studies with other HRQoL instruments could further strengthen our understanding of its utility. Such efforts are expected to enhance the evidence base for adolescent well-being assessment and inform the development of targeted interventions to promote mental health and overall quality of life. Overall, this study advances knowledge in the field of adolescent HRQoL measurement, providing a concise and reliable tool that can support both research and applied practice while guiding future studies aimed at improving adolescent health outcomes in diverse contexts.

## Figures and Tables

**Table 1:** General description of the sample.

Age	n	%
11 to 12 years	274	24.4
13 to 14 years	673	59.9
15 to 17 years	177	15.7
Sex		
Male	523	46.5
Female	601	53.5
Grade		
First year of secondary school	235	20.9
Second year of secondary school	340	30.2
Third year of secondary school	365	32.5
First year of preparatory high school	184	16.4

**Table 2:** Mean, standard deviation, skewness, and kurtosis between the items of the Kidscreen-27.

Dimension/Item	M	SD	Skewness	Kurtosis
Physical Well-Being				
1- In general, how would you say your health is?	3.07	0.95	0.40	−0.34
2- Have you felt fit and well?	3.39	1.03	−0.07	−0.52
3- Have you felt fit and well?	3.68	1.12	−0.38	−0.79
4- Have you been able to run well?	3.90	1.08	−0.68	−0.36
5- Have you felt full of energy?	3.59	1.07	−0.40	−0.45
Psychological Well-Being				
6- Has your life been enjoyable?	3.90	1.04	−0.58	−0.56
7- Have you been in a good mood?	3.55	1.00	−0.41	−0.20
8- Have you had fun?	4.02	0.95	−0.65	−0.31
9- Have you felt sad?	2.96	1.02	−0.08	−0.29
10- Have you felt so bad that you didn’t want to do anything?	3.27	1.16	−0.19	−0.64
11- Have you felt lonely?	3.33	1.26	−0.22	−0.92
12- Have you been happy with the way you are?	3.56	1.25	−0.47	−0.79
Autonomy and Parents				
13- Have you had enough time for yourself?	3.58	1.17	−0.38	−0.77
14- Have you been able to do the things that you want to do in your free time?	3.46	1.19	−0.27	−0.87
15- Have your parent(s) had enough time for you?	3.70	1.17	−0.51	−0.75
16- Have your parent(s) treated you fairly?	4.09	1.06	−1.01	0.25
17- Have you been able to talk to your parent(s) when you wanted to?	3.72	1.32	−0.64	−0.84
18- Have you had enough money to do the same things as your friends?	3.52	1.20	−0.33	−0.80
19- Have you had enough money for your expenses?	3.71	1.14	−0.47	−0.68
Social Support and Peers				
20- Have you spent time with your friends?	3.91	1.09	−0.71	−0.35
21- Have you had fun with your friends?	4.34	0.93	−1.50	1.96
22- Have you and your friends helped each other?	4.15	0.97	−0.97	0.35
23- Have you been able to rely on your friends?	3.79	1.14	−0.60	−0.45
School Environment				
24- Have you been happy at school?	3.67	1.03	−0.50	−0.22
25- Have you got on well at school?	3.73	1.01	−0.51	−0.15
26- Have you been able to pay attention?	3.70	1.03	−0.50	−0.29
27- Have you got along well with your teachers?	3.72	1.05	−0.54	−0.21

Note: distributions with values of skewness and kurtosis between ±2.0 are considered normal.

**Table 3:** Confirmatory factor analysis on the Mexican adolescent sample.

Model	χ^2^ (*df*)		RMSEA	CFI	TLI	NFI	SRMR	χ^2^/*df*
M1 Initial	2050.40	314	0.070	0.847	0.829	0.825	0.0578	6.53
M2 (err9 ↔ err10)	1828.90	313	0.066	0.867	0.851	0.844	0.0548	5.84
M3 (err10 ↔ err11)	1728.10	312	0.064	0.876	0.860	0.853	0.0528	5.53
M4 (err9 ↔ err11)	1575.70	311	0.060	0.889	0.875	0.866	0.0502	5.06
M5 (err18 ↔ err19)	1202.80	310	0.051	0.922	0.911	0.897	0.0463	3.88
M6 (err3 ↔ err4)	1118.64	309	0.048	0.929	0.919	0.905	0.0432	3.62

**Table 4:** Internal consistency coefficients.

	α	ω	M	SD
Physical Well-Being	0.773	0.782	17.61	3.82
Psychological Well-Being	0.803	0.805	24.57	5.24
Autonomy and Relationship with Parents	0.845	0.845	25.78	5.97
Social and Peers Support	0.772	0.772	16.19	3.19
School Environment	0.727	0.735	14.82	3.06

**Table 5:** Correlations between KIDSCREEN-27 dimensions and convergent constructs.

Variable	1	2	3	4	5	6	7	8	9
1. Self-Esteem	1								
2. Subjective Well-Being	0.580 **	1							
3. Satisfaction With Life	0.444 **	0.490 **	1						
4. HRQoL	0.641 **	0.769 **	0.521 **	1					
5. Physical Well-Being	0.454 **	0.588 **	0.345 **	0.740 **	1				
6. Psychological Well-Being	0.652 **	0.695 **	0.461 **	0.824 **	0.553 **	1			
7. Autonomy and Relationship with Parents	0.529 **	0.642 **	0.479 **	0.858 **	0.495 **	0.626 **	1		
8. Social and Peer Support	0.272 **	0.430 **	0.260 **	0.604 **	0.358 **	0.377 **	0.424 **	1	
9. School Environment	0.450 **	0.490 **	0.355 **	0.683 **	0.374 **	0.473 **	0.471 **	0.373 **	1

** *p* < 0.01.

**Table 6:** Correlations between KIDSCREEN-27 dimensions and discriminant constructs.

Variable	1	2	3	4	5	6	7	8	9
1. Perceived Stress	1								
2. Anxious and Depressive Symptomatology	0.648 **	1							
3. Academic Stress	0.456 **	0.489 **	1						
4. HRQoL	−0.606 **	−0.525 **	−0.317 **	1					
5. Physical Well-Being	−0.408 **	−0.317 **	−0.148 **	0.740 **	1				
6. Psychological Well-Being	−0.621 **	−0.571 **	−0.334 **	0.824 **	0.553 **	1			
7. Autonomy and Parents	−0.532 **	−0.431 **	−0.254 **	0.858 **	0.495 **	0.626 **	1		
8. Social and Peer Support	−0.262 **	−0.235 **	−0.124 **	0.604 **	0.358 **	0.377 **	0.424 **	1	
9. School Environment	−0.384 **	−0.400 **	−0.322 **	0.683 **	0.374 **	0.473 **	0.471 **	0.373 **	1

** *p* < 0.01.

**Table 7:** Configural, metric, and scalar measurement invariance across sex.

	*χ*^2^ (*df*)	*p*	CFI	TLI	SRMR	RMSEA	Δ*χ*^2^ (Δ*df*)	*p*	ΔCFI	ΔTLI	ΔSRMR	ΔRMSEA
Sex												
M_1_: Configural	1730.13	0.00	0.89	0.88	0.0541	0.040	-	-	-	-	-	-
M_2_: Metric	1777.38	0.00	0.89	0.88	0.0562	0.040	47.24 (22)	0.00	0.00	0.00	0.0021	0.00
M_3_: Scalar	1788.14	0.00	0.89	0.88	0.0585	0.040	10.78 (5)	0.05	0.00	0.00	0.0023	0.00

## Data Availability

The data presented in this study are available on request from the corresponding author. The data are not publicly available due to the protection of personal data that could compromise the privacy of research participants.

## Abbreviations

The following abbreviations are used in this manuscript:

CFA	Confirmatory Factor Analysis
CFI	Comparative Fit Index
EFA	Exploratory Factor Analysis
HRQoL	Health-Related Quality of Life
KMO	Kaiser–Meyer–Olkin
MG-CFA	Multi Group Confirmatory Factor Analyses
MI	Modification Indices
PHQ-4	Patient Health Questionnaire of 4 items
PSS-10	Perceived Stress Scale of 10 items
QoL	Quality of Life
RMSEA	Root Mean Square Error of Approximation
RSES	Rosenberg Self-Esteem Scale
SRMR	Residual-Based Absolute Fit Index
SWLS	Satisfaction With Life Scale
TLI	Tucker–Lewis Index
WHO	World Health Organization
WHO-5	WHO Well-Being Index
